# A Psychometric Properties Evaluation of the Italian Version of the Geriatric Depression Scale 

**DOI:** 10.1155/2018/1797536

**Published:** 2018-03-01

**Authors:** Giovanni Galeoto, Julita Sansoni, Michela Scuccimarri, Valentina Bruni, Rita De Santis, Mariele Colucci, Donatella Valente, Marco Tofani

**Affiliations:** ^1^Department of Public Health and Infection Disease, “Sapienza” University of Rome, Rome, Italy; ^2^Department of Anatomical, Histological, Forensic and Orthopedic Sciences, “Sapienza” University of Rome, Rome, Italy; ^3^Nursing Home “Ancelle Francescane del Buon Pastore”, Via di Vallelunga 8, 00166 Rome, Italy; ^4^Department of Paediatrics and Child Neuropsychiatry, “Sapienza” University of Rome, Rome, Italy; ^5^Department of Neurosciences and Neurorehabilitation, Bambino Gesù Children's Hospital, Via della Torre di Palidoro, Palidoro, 00054 Rome, Italy

## Abstract

**Objective:**

The Geriatric Depression Scale (GDS) is an evaluation tool to diagnose older adult's depression. This questionnaire was defined by Yesavage and Brink in 1982; it was designed expressly for the older person and defines his/her degree of satisfaction, quality of life, and feelings. The objective of this study is to evaluate the psychometric properties of the Italian translation of the Geriatric Depression Scale (GDS-IT).

**Methods:**

The Italian version of the Geriatric Depression Scale was administered to 119 people (79 people with a depression diagnosis and 40 healthy ones). We examined the following psychometric characteristics: internal consistency reliability, test-retest reliability, concurrent validity, and construct validity (factor structure).

**Results:**

Cronbach's Alpha for the GDS-IT administered to the depressed sample was 0.84. Test-retest reliability was 0.91 and the concurrent validity was 0.83. The factorial analysis showed a structure of 5 factors, and the scale cut-off is between 10 and 11.

**Conclusion:**

The GDS-IT proved to be a reliable and valid questionnaire for the evaluation of depression in an Italian population. In the present study, the GDS-IT showed good psychometric properties. Health professionals now have an assessment tool for the evaluation of depression symptoms in the Italian population.

## 1. Introduction

The Geriatric Depression Scale (GDS) is one of the most popular scales for the evaluation of depression symptoms in older adults; it is composed of 30 items and excludes somatic and psychotic symptoms. Items are scored dichotomous and this fact makes the tool easier to use in older patients with cognitive deficits [1]. The GDS is defined self-report and needs an average of 20 minutes to complete. The score goes from 0 (not depressed) to 30 (serious depression) with a cut-off at score 11 in the original version. In the literature, there are many studies that described the validation of the GDS either in the original formula or the short ones with 5, 10, and 15 items.

According to a review in 2006, the average of the scale's sensitivity is 0.753 while the specificity's one is 0.770, which is considered modest but not excellent. Among the 33 studies included in the review, most studies declare 10 (8 studies) or 11 (13 studies) as the cut-off; just two studies state a cut-off lower than 7 [2].

The GDS-30 was validated in Chinese [3], Greek [4], Spanish [5], Korean [6], Portuguese [7], and Singhalese [8]. It was also validated for different pathologies: adults who are poststroke [9], in palliative care [10], who have Parkinson's disease [11], and who have Alzheimer's disease [12].

In Italy, the GDS is widely used but all the psychometric properties have not been investigated. Considering that in Italy depression is one of the most common mental disorders [13]; it seems necessary to validate an assessment tool for the screening of depression. Therefore, the objective of the present study is to evaluate the psychometric properties of the Italian translation of the GDS through a cross-sectional study.

## 2. Methods

After having received the confirmation for the realization of the study from the authors of the original version and the authorization from Professor Fabiano Cavarzeran for the use of the Italian translation of the instrument, the research protocol was drafted as recommended by international guidelines [14]. In order to draw up the protocol, we followed the original version that considered the validation of the instrument on a sample of adults with depression and healthy people.

Between November 2015 and March 2016, the sample of individuals with a diagnosis of depression was recruited from four clinics in the urban area of Rome. Before administering the test, each person was informed about the study and they signed an informed consent [15, 16]. All the participant had to respect the following criteria: age ≥ 65, MMSE ≥ 18/30 [17], and a confirmed diagnosis of depression, according to the Diagnostic and Statistical Manual of Mental Disorders, Fourth Edition, Text Revision (DSM-IVTR) (American Psychiatric Association, 2000). The inclusion criteria for the health group were age ≥ 65, MMSE ≥ 18/30 [17].

For the test-retest reliability, 30 people with depression diagnoses were randomly selected from the study sample. The GDS-IT was administered twice at a maximum distance of 6 days. For a significant statistical value, the scale was considered stable for ICC > 0.70. The internal consistency of the GDS-IT was examined by Cronbach's Alpha in order to assess the correlation of the item and the homogeneity of the scale. The limit was set a 0.60. To determine the concurrent validity, we compared the obtained score on the GDS-IT to a diagnosis of depression according to DSM-IV TR.

The appropriateness of sampling was evaluated using the Keiser-Meyer-Olkin (KMO) and Bartlet test. The factorial structure of the test was determined through the analysis of the principal components with oblique rotation and with the maximum likelihood solution. It was made according to Graetz's recommendations, who states that, with the oblique rotation, results are more convenient and provide an easily interpreting solution.

To compare our test and depression diagnosis' data, the ROC curve and the area under the AUC's one were created and valuated. Collectively, a 1.0 AUC refers to a precise data, while an imprecise one shows a 0.0 AUC. Usually, an AUC higher than 0.75 shows that scale predictors are moderate, while the excellent ones are obtained with an AUC ≥ 0.90. The best cut-off point was chosen to give the maximum Youden Index [18]. Two *p* < 0.05 were considered statistically significant. The agreement measure among the register's score and scale's one was evaluated through the Kappa test. The acceptability of the scale was evaluated in terms of time, multiple entries, and compilation of misprints. All the statistical analyses were carried out with the statistical package of Social Sciences (SPSS), 18.0 Windows version.

## 3. Results

### 3.1. Participants

**Figure 1:**
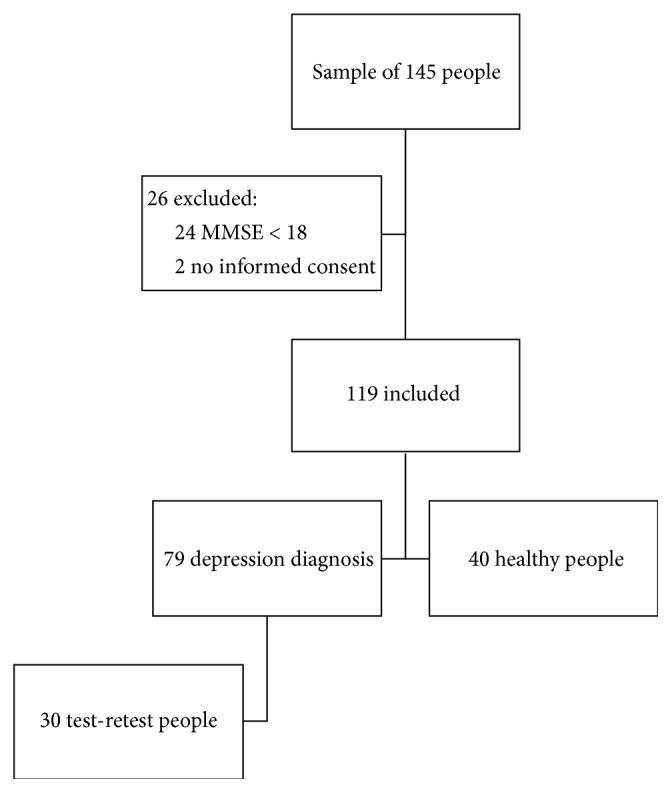
Flowchart.

For our study, 145 people were evaluated, where 119 respected the criteria for the inclusion. 40 healthy people were recruited from community setting. In Figure 1, there is the sample of healthy and pathological people. The characteristics of the sample can be found in Table 1.

### 3.2. Reliability Test (Test Retest)

30 people recruited for the test-retest interrater showed statistically significant results for every item with an ICC higher than 0.60 and an ICC of 0.91 for the whole scale (Table 2).

### 3.3. Internal Consistency and Reliability

Internal consistency and reliability were evaluated through the correlation of Pearson and Cronbach's Alpha. An item for item correlation and total item of them (Table 3) showed statistically significant data. Cronbach's Alpha scored 0.839.

### 3.4. Factorial Analysis

The Bartlett test showed a value of 0.563 and with *p* < 0.001; this shows a good suitability of the sample. The factorial analysis produced 5 factors for the GDS-IT that represents the 54.7% of the variance (Table 4).

The first factor is composed of 8 items (“Are you bothered by thoughts you can't get out of your head?” “Are you afraid that something bad is going to happen to you?” “Do you often feel helpless?” “Do you often get restless and fidgety?” “Do you feel downhearted and blue?” “Do you feel that your situation is hopeless?” “Do you frequently get upset over little things?” “Do you frequently feel like crying?”) that contribute to 17.89% of the total of variance. This factor is called “sad mood and agitation.”

The second factor is composed of 7 items (“Are you basically satisfied with your life?” “Do you feel that your life is empty?” “Do you think it is wonderful to be alive now?” “Do you feel pretty worthless the way you are now?” “Do you find life very exciting?” “Do you think that most people are better off than you are?” “Is your mind as clear as it used to be?”) that contribute to 12.72% of the total of variance. This factor is called “cognitive inefficiency.”

The third factor is composed of 4 items (“Have you dropped many of your activities and interests?” “Is it hard for you to get started on new projects?” “Do you feel full of energy?” “Is it easy for you to make decisions?”) that contribute to 9.78% of the variance. This factor is called “lack of energy.”

The forth factor is composed of 4 items (“Are you hopeful about the future?” “Are you in good spirits most of the time?” “Do you feel happy most of the time?” “Do you enjoy getting up in the morning?”) that contribute to 7.52% of the variance. This factor is called “positive mood.”

The fifth factor is composed of 7 items (“Do you often get bored?” “Do you prefer to stay at home, rather than going out and doing new things?” “Do you frequently worry about the future?” “Do you feel you have more problems with memory than most?” “Do you worry a lot about the past?” “Do you have trouble concentrating?” “Do you prefer to avoid social gatherings?”) that contribute to 6.76% of the variance. This factor is called “social withdrawal.”

### 3.5. Prognosis

**Figure 2:**
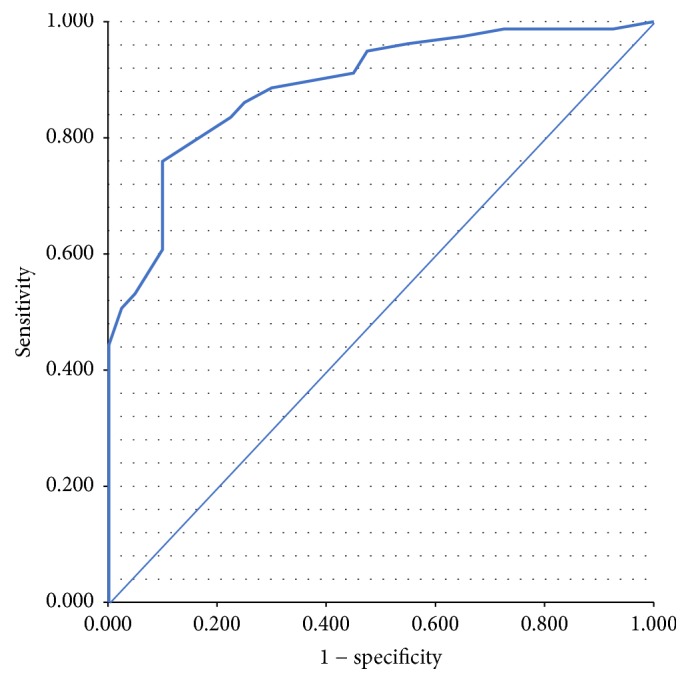
*ROC curve.* Diagonal segments are produced by ties.

Regarding the evaluation of patients with depression, the area under the curve (AUC) showed a value of 0.901 (CI 0.739–0.811 95%) (Figure 2). The maximized score that predicts depression is between 10/11 (sensitivity, 84%; specificity, 77.5%).

The Kappa test, for the agreement between the measure of the scale and the register for limitations from manual handling of loads, showed an agreement of 0.42 with *p* < 0.0001.

### 3.6. Acceptability

The average of compilation was 10.6 ± 3.3 minutes in the first revelation (2–13 range) and 9.7 ± 2.9 in the second one (2–12 range). There were no double reactions or misprints.

## 4. Discussion

The depression is a pathology that respects specific criteria to make a diagnosis but the evaluation and the resolution of problems correlated to that pathology are not so easy as well.

The GDS-30 has been used and in previous two Italian studies in Alzheimer disease, it has not undergone a validation process yet [19, 20].

The objective of this study was to verify that the GDS-30 is a valid tool that can be used to evaluate the psychoemotional status of patients with depression.

The validation process of the Italian translation of the Geriatric Depression Scale (GDS-IT) showed statistically significant data regarding the reliability and internal consistency of the GDS-IT. Thus, all of the items are correlated and Cronbach's Alpha has a value of 0.839, in line with the Spanish version [5], but slower lighter than Portuguese [7] and original [1] validation studies. This data shows that the scale results are reliable and have a good internal consistency.

In the study, the cut-off score of the GDS-IT in patients with depression is 10/11, with sensitivity at the 84%, and a specificity at the 77.5%. This data is consistent with the English (cut-off 10/11), Portuguese (cut-off 11), and Spanish (cut-off 9/10) studies and with most of the studies of validation of the Geriatric Depression Scale [2].

The GDS-IT factorial analysis showed a structure with 5 factors almost similar to the structure of the original version [21]. The original version is composed of 5 factors: sad mood (8, 6, 23, 13, 15, 18, 10, 24, 22), lack of energy (29, 20, 21, 30, 25, 2), positive mood (15, 27, 9, 5, 7, 19), agitation (24, 11, 4), and social withdrawal (12, 28). From the original version 4 items stayed out. The factorial analysis that we obtained is also similar to the Korean version of the Geriatric Depression Scale. This one is composed of 5 factors: sad mood and agitation (6, 18, 11, 8, 13, 24, 16, 25, 10, 3), positive mood (1, 9, 7, 15, 19, 22, 27, 5, 23), lack of energy (2, 21, 20, 17), cognitive inefficiency (14, 26, 30), and social withdrawal (12, 28) [6].

In the English version, 4 items do fall under any factor and they are 1, 3, 14, and 17. In the GDS-IT, items “Are you basically satisfied with your life?” “Do you feel that your life is empty?” “Do you feel pretty worthless the way you are now?” are included in factor 2, while the item “Do you feel you have more problems with memory than most?” is included in factor 5.

In the Korean version, item 29 was excluded by the factorial analysis that in the GDS-IT is in factor 3. Differences between the factorial analysis of the mentioned versions (English and Korean) could be cultural and attributed to samples of study among the various validations.

At the end of the study, the GDS-IT revealed itself as easy to use and understand and, according to the obtained results, has been shown to be valid and reliable tool in Italian population and is recommended for use in clinical practice. However, to better understand the complexity of the target population, a clinical investigation in the most common principal diagnoses of the elderly people with a larger sample size is required.

## Figures and Tables

**Table 1:** Characteristics of the sample.

	Depression diagnosis	Healthy
Sample	79	40
Age (average ± DS)	76,6 ± 8,2	74,8 ± 8,5
Sex F *n* (%)	55 (69,6%)	18 (45%)
MMSE (average ± DS)	24,28 ± 3,4	26,93 ± 2,4

**Table 2:** Test-retest analysis.

Item	Test	Retest	ICC	IC 95%
	M (DS)	M (DS)		
(1)	0,53 ± 0,50	0,57 ± 0.50	0,935	(0,868–0,968]
(2)	0,50 ± 0,50	0,47 ± 0,50	0,668	(0,410–0,827]
(3)	0,53 ± 0,50	0,60 ± 0,49	0,873	(0,750–0,937]
(4)	0,70 ± 0,46	0,80 ± 0,40	0,757	(0,549–0,876]
(5)	0,73 ± 0,45	0,77 ± 0,43	0,736	(0,516–0,865]
(6)	0,73 ± 0,45	0,77 ± 0,45	0,689	(0,162–0,719]
(7)	0,57 ± 0,50	0,67 ± 0,47	0,808	(0,634–0,904]
(8)	0,33 ± 0,47	0,27 ± 0,45	0,532	(0,218–0,746]
(9)	0,57 ± 0,50	0,57 ± 0,50	0,729	(0,504–0,861]
(10)	0,67 ± 0,47	0,70 ± 0,46	0,663	(0,129–0,702]
(11)	0,80 ± 0,40	0,73 ± 0,45	0,638	(0,365–0,809]
(12)	0,50 ± 0,50	0,53 ± 0,50	0,668	(0,410–0,827]
(13)	0,43 ± 0,50	0,40 ± 0,49	0,659	(0,397–0,822]
(14)	0,33 ± 0,47	0,37 ± 0,49	0,782	[0,592–0,890]
(15)	0,20 ± 0,40	0,23 ± 0,43	0,611	[0,191–0,733]
(16)	0,60 ± 0,49	0,63 ± 0,49	0,649	[0,383–0,816]
(17)	0,47 ± 0,50	0,47 ± 0,50	0,866	[0,738–0,934]
(18)	0,30 ± 0,46	0,67 ± 0,47	0,782	[0,592–0,890]
(19)	0,40 ± 0,49	0,43 ± 0,50	0,934	[0,865–0,968]
(20)	0,63 ± 0,49	0,70 ± 0,46	0,558	[0,253–0,762]
(21)	0,47 ± 0,50	0,70 ± 0,46	0,61	[0,326–0,793]
(22)	0,30 ± 0,46	0,30 ± 0,46	0,683	[0,432–0,835]
(23)	0,43 ± 0,50	0,50 ± 0,50	0,874	[0,735–0,938]
(24)	0,70 ± 0,46	0,67 ± 0,47	0,925	[0,850–0,954]
(25)	0,600 ±,49	0,57 ± 0,50	0,934	[0,865–0,968]
(26)	0,23 ± 0,46	0,33 ± 0,47	0,609	[0,325–0,793]
(27)	0,60 ± 0,49	0,57 ± 0,50	0,659	[0,397–0,822]
(28)	0,33 ± 0,47	0,25 ± 0,47	0,691	[0,446–0,840]
(29)	0,40 ± 0,49	0,37 ± 0,49	0,932	[0,862–0,967]
(30)	0,60 ± 0,49	0,60 ± 0,49	0,861	[0,729–0,931]

Total	15,37 ± 5,46	16,17 ± 5,76	0,914	[0,828–0,958]

**Table 3:** Item X tot-item analysis.

Item	Corrected item-total correlation	Cronbach's Alpha if item was deleted	Corrected item-total correlation	Cronbach's Alpha if item was deleted
(1)	,104	,791	,597	,809
(2)	,009	,795	−,075	,836
(3)	,256	,784	,236	,821
(4)	,335	,780	,472	,812
(5)	,445	,775	,173	,823
(6)	,361	,779	,426	,814
(7)	,469	,773	,180	,822
(8)	,469	,773	,331	,818
(9)	,325	,780	,457	,813
(10)	,517	,771	,257	,820
(11)	,399	,777	,683	,801
(12)	,183	,787	,090	,827
(13)	,203	,786	,530	,809
(14)	,289	,782	,395	,817
(15)	,258	,783	,000	,824
(16)	,594	,767	,409	,815
(17)	,271	,783	,568	,812
(18)	,171	,787	,597	,806
(19)	,129	,789	,506	,812
(20)	,295	,782	,481	,811
(21)	,212	,785	,182	,824
(22)	,443	,775	,451	,816
(23)	,443	,775	,196	,821
(24)	,480	,773	,474	,811
(25)	,440	,775	,318	,818
(26)	,305	,781	,120	,825
(27)	,140	,789	,250	,820
(28)	−,065	,796	,491	,813
(29)	,365	,778	,020	,830
(30)	,004	,794	,318	,818

**Table 4:** Factorial analysis.

	Factor				
	1	2	3	4	5
(1) Are you basically satisfied with your life?		**0,62**			
(2) Have you dropped many of your activities and interests?			**0,63**		
(3) Do you feel that your life is empty?		**0,51**			
(4) Do you often get bored?					**0,53**
(5) Are you hopeful about the future?				**0,56**	
(6) Are you bothered by thoughts you can't get out of your head?	**0,57**				
(7) Are you in good spirits most of the time?				**0,6**	
(8) Are you afraid that something bad is going to happen to you?	**0,75**				
(9) Do you feel happy most of the time?				**0,70**	
(10) Do you often feel helpless?	**0,69**				
(11) Do you often get restless and fidgety?	**0,65**				
(12) Do you prefer to stay at home, rather than going out and doing new things?					**0,41**
(13) Do you frequently worry about the future?					**0,38**
(14) Do you feel you have more problems with memory than most?					**0,64**
(15) Do you think it is wonderful to be alive now?		**0,36**			
(16) Do you feel downhearted and blue?	**0,78**				
(17) Do you feel pretty worthless the way you are now?		**0,86**			
(18) Do you worry a lot about the past?					**0,62**
(19) Do you find life very exciting?		**0,78**			
(20) Is it hard for you to get started on new projects?			**0,63**		
(21) Do you feel full of energy?			**0,53**		
(22) Do you feel that your situation is hopeless?	**0,61**				
(23) Do you think that most people are better off than you are?		**0,52**			
(24) Do you frequently get upset over little things?	**0,63**				
(25) Do you frequently feel like crying?	**0,67**				
(26) Do you have trouble concentrating?					**0,34**
(27) Do you enjoy getting up in the morning?				**0,51**	
(28) Do you prefer to avoid social gatherings?					**0,31**
(29) Is it easy for you to make decisions?			**0,65**		
(30) Is your mind as clear as it used to be?		**0,25**			

Percent of variance%	17,89	12,72	9,78	7,52	6,76
